# Altered lipid composition in *Streptococcus pneumoniae cpoA* mutants

**DOI:** 10.1186/1471-2180-14-12

**Published:** 2014-01-20

**Authors:** Marina Meiers, Carsten Volz, Jessica Eisel, Patrick Maurer, Bernhard Henrich, Regine Hakenbeck

**Affiliations:** 1 Department of Microbiology, University of Kaiserslautern, Gottlieb-Daimler-Strasse, Gebäude 23, D-67663 Kaiserslautern, Germany; 2 Present address: Department of Microbial Natural Products, Helmholtz-Institute for Pharmaceutical Research Saarland (HIPS), Saarland University, D-66123 Saarbrücken, Germany; 3 Present address: Hochschule für Technik und Wirtschaft des Saarlandes, Goebenstrasse 40, D-66117 Saarbrücken, Germany

**Keywords:** *Streptococcus pneumoniae*, Glycolipids, Penicillin resistance, Glycosyltransferase, CpoA, Phospholipid

## Abstract

**Background:**

Penicillin-resistance in *Streptococcus pneumoniae* is mainly due to alterations in genes encoding the target enzymes for beta-lactams, the penicillin-binding proteins (PBPs). However, non-PBP genes are altered in beta-lactam-resistant laboratory mutants and confer decreased susceptibility to beta-lactam antibiotics. Two piperacillin resistant laboratory mutants of *Streptococcus pneumoniae* R6 contain mutations in the putative glycosyltransferase gene *cpoA*. The CpoA gene is part of an operon including another putative glycosyltransferase gene *spr0982*, both of which being homologous to glycolipid synthases present in other Gram-positive bacteria.

**Results:**

We now show that the *cpoA* mutants as well as a *cpoA* deletion mutant are defective in the synthesis of galactosyl-glucosyl-diacylglycerol (GalGlcDAG) *in vivo* consistent with the *in vitro* function of CpoA as α**-**GalGlcDAG synthase as shown previously. In addition, the proportion of phosphatidylglycerol increased relative to cardiolipin in *cpoA* mutants. Moreover, *cpoA* mutants are more susceptible to acidic stress, have an increased requirement for Mg^2+^ at low pH, reveal a higher resistance to lysis inducing conditions and are hypersensitive to bacitracin.

**Conclusions:**

The data show that deficiency of the major glycolipid GalGlcDAG causes a pleitotropic phenotype of *cpoA* mutant cells consistent with severe membrane alterations. We suggest that the *cpoA* mutations selected with piperacillin are directed against the lytic response induced by the beta-lactam antibiotic.

## Background

Development of resistance to beta-lactam antibiotics in *Streptococcus pneumoniae* involves alterations in the target proteins, the penicillin-binding proteins (PBPs) which result in decreased affinity to beta-lactams. In order to identify individual mutations in *S. pneumoniae* that are related to the resistance phenotype, a series of independent mutant families has been selected in the laboratory using stepwise increasing concentrations of antibiotics [1]. Two beta-lactams were chosen for selection: piperacillin, which induces rapid lysis in the bacteria, and cefotaxime which does not interact with PBP2b and leads to a tolerant response [2]. Point mutations in *pbp2b* from piperacillin-resistant mutants and in *pbp2x* from cefotaxime resistant mutants have been described [3-5]. Surprisingly, a decrease in antibiotic susceptibility in some mutants correlated with a mutation in non-PBP genes [6]. In two piperacillin-resistant mutants, P106 and P104, obtained independently after the first selection step before the introduction of PBP mutations, the putative glycosyltransferase (GT) gene *cpoA* was affected [7]. Decreased susceptibility for piperacillin of the *cpoA* mutants was accompanied by a pleiotropic phenotype such as a defect in genetic competence and reduced amount of PBP1a. This indicated a novel mechanism directed against the activity of lytic β-lactams in *S. pneumoniae* distinct from target-mediated resistance.

The CpoA gene *spr0981* and the adjacent gene *spr0982* encode putative GTs which belong to the GTB-type superfamily (GT1-YqgM-like family). Members of this GT family are anchored in the membrane cytoplasmic interface by hydrophobic and charge interactions [8,9] and transfer a sugar moiety to an acceptor molecule located in the inner leaflet of the membrane. Therefore, it had been proposed that CpoA perfoms a similar function in *S. pneumoniae*[7]. Meanwhile, *in vitro* studies revealed that both proteins are involved in the synthesis of glycolipids, with Spr0982 acting as α-monoglucosyl-diacylglycerol (GlcDAG) synthase and CpoA as a α-galactosyl-glucosyl-diacylgylcerol (GalGlcDAG) synthase [9,10]. These two glycolipids occur at a ratio of approximately 1:2.5 in the *S. pneumoniae* membrane [11], in addition to phosphatidyl glycerol and cardiolipin which constitute the major phospholipids [12].

By consecutively synthesizing one nonbilayer-prone (mono-glucosyl-DAG) and one bilayer-forming glycolipid (di-glycosyl-DAG), the function of the GTs is crucial for the bilayer spontaneous curvature which affects the physical properties of the cytoplasmic membrane [13]. An example is the mycoplasma *Acholeplasma laidlawii*, where bilayer curvature is extensively regulated by two closely related GTs consecutively synthesizing monoglucosyl-DAG and diglucosyl-DAG [9,13], enzymes that are homologous to *S. pneumoniae* Spr0982 and CpoA. Thus it is most likely that CpoA and Spr0982 play a critical role in *S. pneumoniae* related to membrane associated functions in agreement with the pleiotropic phenotype of the CpoA mutants mentioned above. GlcDAG is the proposed lipid anchor of the essential choline-containing lipoteichoic acid (LTA) of *S. pneumoniae*[14]. In fact, *spr0982* has been listed among essential genes of this organism [15].

In the present report, a *cpoA* deletion mutant was constructed and compared to the CpoA mutants P106 and P104; moreover, the *cpoA* operon was investigated by mutational analysis. The aim of this study was to examine the function of CpoA *in vivo*, and to further our understanding on the physiological consequences of *cpoA* mutations.

## Results

### The CpoA gene is part of an operon with five downstream genes

**Figure 1:**
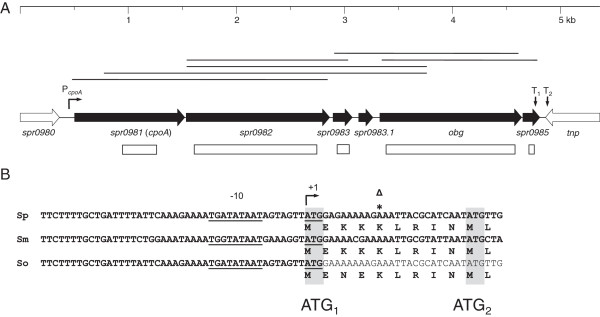
**Genes, transcription and deletions in the *cpoA-spr0985 *region of *S. pneumoniae *R6. (A)** Wide horizontal arrows indicate genes apparently co-transcribed with *cpoA* (black), and flanking genes (white). *spr0983.1* has not been annotated in the R6 genome [20], but its presence has been predicted from other *S. pneumoniae* genomes such as TIGR4 [56]. The positions and extend of in-frame deletions are shown as white boxes below the respective genes. Lines above the genetic map represent DNA products obtained by RT-PCR with total RNA and gene-specific primers. The positions of the promoter P_*cpoA*_ and of putative ρ-independent terminators (T_1_ [ΔG = −10.4 kcal/mol], T_2_ [ΔG = −10.1 kcal/mol]) are given by angled and vertical arrows, respectively. **(B)** The nucleotide sequence upstream of *S. pneumoniae* R6 *cpoA* and putative 3'-coding sequences is shown together with the predicted peptide sequence (Sp). The −10 element of P_*cpoA*_ is underlined, and the transcription start site (+1) is indicated with an angled arrow. The position of an adenine nucleotide, deleted in the mutant strain P106 [7] is marked with *Δ. Two potential start codons of the *cpoA* gene (ATG_1_, ATG_2_; see text for detail) are underlined. The respective *cpoA* sequences of *S. mitis* B6 (Sm) and *S. oralis* Uo5 (So) are shown below.

P104 and P106 are spontaneous piperacillin-resistant laboratory mutants isolated independently after one selection step from the laboratory strain *S. pneumoniae* R6 [4,7]. Both mutants contain a mutation affecting CpoA: in P104, a transversion within *cpoA* GTA to GGA led to a Gly12Val exchange in the predicted protein product, whereas in P106, one adenine nucleotide was deleted 15 base pairs (bp) upstream of the proposed *cpoA* start codon (ATG_2_ in Figure 1) [7]. Although ATG_2_ is not preceded by a classical Shine Dalgarno sequence, this deletion was suspected to affect the efficiency of ribosome binding to the *cpoA* transcript [7]. However, the possibility remained that translation actually starts at an alternative start codon (ATG_1_ in Figure 1) 27 bp upstream of ATG_2_ which is preceded by a perfect −10 region. In this case, the deletion in P106 would lead to a frameshift in the 5th codon and thus to the production of a nonsense peptide.

To first clarify this issue, the expression signals of *cpoA* were mapped. The 5' end of *cpoA* mRNA was determined by RACE, and shown to be located 27 bp upstream of ATG_2_ (Figure 1B). Since this is exactly the position of the alternative start codon ATG_1_, translation initiation at ATG_1_ would imply that the *cpoA* transcript is leaderless [16]. In order to see whether ATG_1_ is indeed functional or whether ATG_2_ is required for translation, three plasmids were constructed in which the inferred promoter P_*cpoA*_ together with either both, ATG_1_ and ATG_2_ (P_*cpoA*_-ATG_12_), ATG_1_ plus a mutated ATG_2_ (P_*cpoA*_-ATG_1_ATA_2_), or ATG_1_ only (P_*cpoA*_-ATG_1_), was translationally fused with the *lacZ* reporter gene. After single-copy integration of the resulting reporter constructs at the *bgaA* locus of R6, the expression of *lacZ* was determined in two transformants in up to three experiments. Beta-galactosidase activity was similar in R6P_*cpoA*_-ATG_1_ATG_2_ and R6P_*cpoA*_-ATG_1_ATA_2_ (190–330 Miller Units), and slightly lower in R6P_*cpoA*_-ATG_1_ containing a shorter region upstream of *lacZ* (140–150 Miller Units), clearly documenting that ATG_1_ is the translation initiation site of *cpoA* and that the *cpoA* transcript is indeed leaderless. In this case, P106 contains a deletion within the structural gene resulting in a frameshift within the 5th codon consistent with the failure to detect CpoA in P106 with a specific anti-CpoA antiserum [7], and the mutation in P104 is Gly21Val.

Comparison with the genetic organization of *cpoA* and upstream regions of the closely related species *S. mitis* B6 and *S. oralis* Uo5 of known genome sequence [17,18] revealed an almost perfect conservation of *cpoA* including the −10 region in these species (Figure 1B).

The arrangement of genes and expression signals predicted in the downstream region of P_*cpoA*_ suggested a polycistronic mRNA of approximately 4.4 kb covering the *cpoA-spr0985* region. This was confirmed by RT-PCR experiments in which six overlapping products were obtained from this region, the largest of which extended from *cpoA* to *spr0984* (Figure 1). Attempts to detect a contiguous transcript of the entire *cpoA*-*spr0985* region, either by RT-PCR or by Northern blot analysis, however, were not successful, probably due to instability of the transcript.

The operon structure of the *cpoA*-*spr0985* region and bioinformatic analyses indicated that the gene products might be functionally related and involved in membrane-associated functions. The GT-activities of CpoA and Spr0982 have been linked to glycolipid biosynthesis by *in vitro* experiments [9,10], Spr0983 [58 amino acids 7(aa)] belongs to the PspC superfamily of putative stress-responsive transcriptional regulators, and Obg (436 aa) belongs to the Obg subfamily of GTP-binding proteins involved in stress response and processes related to cell division [for review, see [19]]. Possible functions of the two small peptides Spr0983.1 (44 aa) which has not been annotated in the R6 genome and Spr0985 (52 aa) [20] cannot be deduced from the amino acid sequences.

### Mutational analysis of the *cpoA* operon

To assess the importance of these gene products, we aimed to construct deletions in each gene. A previous attempt to delete *cpoA* by insertion-duplication mutagenesis using a non-replicative plasmid vector had been unsuccessful [7]. This suggested that either *cpoA* is essential, or that insertion of the vector had affected the expression of the downstream gene *spr0982* which has been listed among essential genes of *S. pneumoniae*[15]. To avoid such polar effects, a different deletion strategy was applied which was based on the construction of in-frame deletions using the Janus cassette (Figure 1). R6 mutants in which 108 central codons of *cpoA* (specifying the GT domain) were replaced with the Janus cassette were obtained with common efficiencies (0.2%), demonstrating that *cpoA* is a non-essential gene. Deletions in *spr0983* and *spr0985* were also obtained. However, the generation times of R6ΔcpoA and R6Δspr0985 (with 46–48 min) were significantly longer compared to R6 and R6Δ*spr0983* (38 min), suggesting that CpoA and Spr0985 are involved in important functions. In contrast, transformants carrying deletions in *spr0982* and *obg* occurred only at 1,000- and respectively 10,000-fold reduced frequencies. This is in agreement with an essential function of the *spr0982* product as reported previously [15], and strongly suggested that also *obg* is indispensable. The rare recovery of transformants carrying deletions in these genes probably was the result of co-selection of compensatory mutations at unknown secondary sites.

### Mutants in *cpoA* are defective in synthesis of diglycosyl-DAG

**Figure 2:**
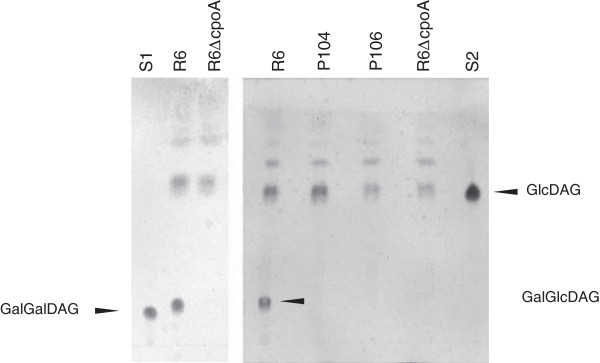
**Glycolipids in Δ*cpoA *and piperacillin resistant laboratory mutants containing *cpoA *mutations.** Lipids extracted from strain R6 and from *cpoA* mutants, P104, P106, and R6ΔcpoA as indicated above the lanes were separated by thin layer chromatography (chloroform/methanol/acetic acid = 80:15:8). GalGalDAG (S1) and GlcDAG (S2) were used as a standards. Spots were assigned to the two major glycolipids of *S. pneumoniae* diglycosyl DAG (GalGlcDAG) and monoglycosyl DAG (GlcDAG).

To verify the CpoA function *in vivo*, the membrane lipids of *cpoA* mutant strains and the parent *S. pneumoniae* R6 were isolated and glycolipids specifically stained after separation by thin layer chromatograpy (Figure 2). *S. pneumoniae* contains the two glycolipids GlcDAG and GalGlcDAG. Two spots were detected in the R6 strain that could be assigned to the pneumococcal glycolipids according to the glycolipid standards: the major one representing a diglycosyl-DAG (most likely GalGlcDAG close to the position of the GalGalDAG standard), and a second spot at the position of monoglycosyl-DAG (Figure 2). This is in agreement with a ratio of GlcDAG to GalGlcDAG to be approximately 1:2.5 [11]. In contrast, the only glycolipid in all *cpoA* mutants corresponded to the position of the monoglycosyl-DAG (Figure 2). This confirms that CpoA is required for the synthesis of the diglycosyl-DAG in *S. pneumoniae* in agreement with the *in vitro* GalGlcDAG-synthase activity of CpoA, and documents that both mutants, P104 and P106, do not contain a functional CpoA.

### Phospholipids in *cpoA* mutants

**Figure 3:**
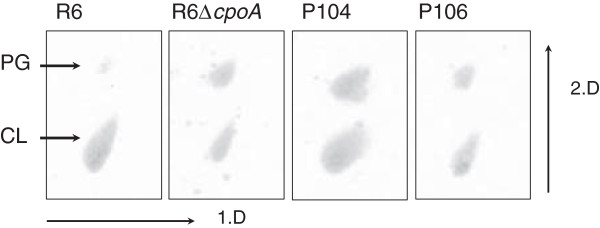
**Phospholipids in *cpoA *mutants.** Lipids were extracted and separated by two dimensional TLC. 1.D and 2.D: first and second dimension (first dimension: CHCl_3_/MeOH/H_2_0 = 65:25:4; second dimension: CHCl_3_/AcOH/MeOH/H_2_0 = 80:14:10:3). Phospholipids were visualized by spraying with Molybdenum Blue spray reagent. PG: phosphatidylgylcerol; CL: cardiolipin. Spots were assigned according to the phosphatidylglycerol standard (see Additional file 1: Figure S1) and Fischer [42].

The glycolipid content affects physical properties of the cytoplasmic membrane. Since the exclusive production of the monolayer-forming glycolipid GlcDAG which forms non-bilayer structures strongly affects the membrane curvature [9,13], we investigated whether this has some impact on the phospholipid content as well. *S. pneumoniae* contains the two phospholipids cardiolipin, a non-bilayer prone lipid, and phosphatidylglycerol. Lipids were separated by two-dimensional thin layer chromatography, and experiments were performed with at least two independently grown cultures. All *cpoA* mutants (R6Δ*cpoA*, P104 and P106) showed a significant increase in the ratio of phosphatidylglycerol: cardiolipin (Figure 3), suggesting that the cells are able to regulate the overall content of bilayer versus non-bilayer forming lipids. It should be noted that phosphadidylglycerol is more strongly stained compared to cardiolipin by the procedure used here (Additional file 1: Figure S1).

### Pleiotropic phenotype of *cpoA* mutants

The severe changes in membrane lipids in *cpoA* mutants is consistent with their pleiotropic phenotype described before [1,7] which included a reduced generation time in liquid medium, decreased susceptibility to beta-lactams, defects in transformability, and a lower amount of PBP1a with less than 20% compared to the parental strain while the *pbp1a* transcript was unaffected; alterations in other PBPs were not detected. We first verified these properties for the R6Δ*cpoA* mutant: the MIC of piperacillin increased from 0.015 μg/ml (R6) to 0.045 μg/ml, the competence for genetic transformation was approximately 20-fold lower and shifted to the early exponential phase compared to R6, and the amount of PBP1a was decreased (not shown). These phenotypes are reminiscent of those displayed by P104/P106 but were more pronounced in R6Δ*cpoA*, probably a result of the *rpsL* allele.

**Figure 4:**
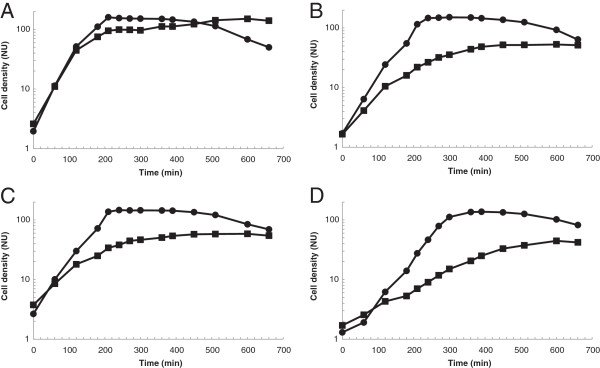
**Growth of *cpoA *mutants in low pH medium.** Strains were grown in C-medium, and culture density was monitored by nephelometry [NU]. The growth was examined at pH 8 (circles) and pH 6 (squares). **A**: R6; **B**: P104; **C**: P106; **D**: R6Δ*cpoA*.

**Figure 5:**
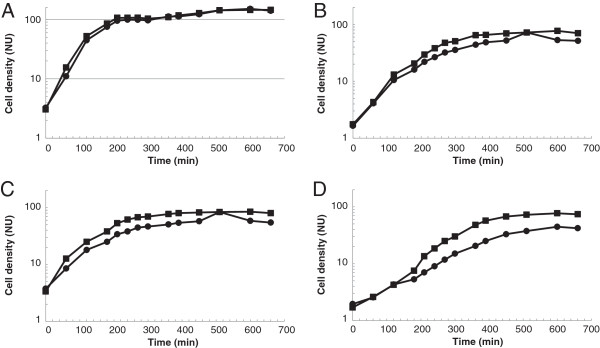
**Mg**^**2+ **^**requirement of *cpoA *mutations.** Strains were grown in C-medium pH 6, and culture density was monitored by nephelometry [NU]. The medium contained either 0.195 mg/ml MgCl_2_ final concentration (filled circles) or 0.39 mg/ml MgCl_2_ (squares). **A**: R6; **B**: P104; **C**: P106; **D**: R6Δ*cpoA*.

**Figure 6:**
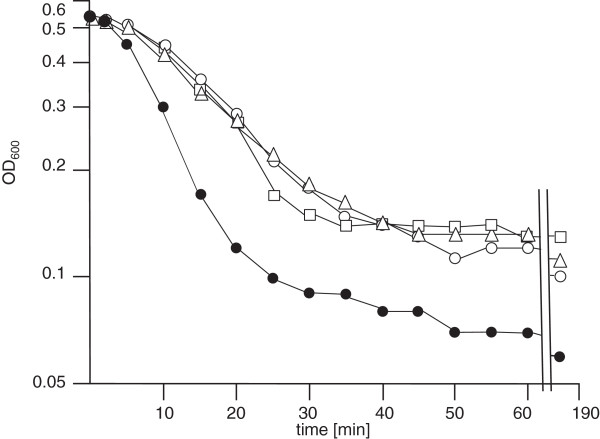
**Triton induced lysis.** Cells were grown to OD_600_ in C-medium. At OD_600_ = 0.5, Triton (0.01% final concentration) was added. R6: filled circles; R6Δ*cpoA*: open circles; P106: open triangles; P104: open squares.

Several other tests were then performed in order to see whether the altered glycolipid composition affects also cell envelope related properties in general. These included growth at low pH, the requirement for Mg^2+^, stationary phase autolysis and lysis induced by Triton X100. In all experiments, *cpoA* mutants showed a clear phenotype distinct from the R6 strain. Growth was severely affected at pH 6 (Figure 4). At pH 6, *cpoA* mutants showed an increased requirement for Mg^2+^ (Figure 5). The stationary phase lysis was slightly delayed in all *cpoA* mutants (Figure 4). Moreover, lysis induced by low concentrations of Triton X100 proceeded significantly more slowly in all *cpoA* mutants (Figure 6).

Susceptibility to non-beta lactam cell wall antibiotics was also tested. Almost no effect was seen with vancomycin (MIC = 0.35 - 0.45 μg/ml) or cycloserine (MIC = 65–75 μg/ml), but the MIC value for bacitracin dropped from 7.5 μg/ml in the R6 strain to 0.75 - 1 μg/ml in all *cpoA* mutants.

### Transcription profile of *cpoA* mutants

The pleiotropic effect of *cpoA* mutants on many membrane-associated functions was consistent with the relation of CpoA activity to glycolipid biosynthesis. In order to estimate the consequences of the altered glycolipid composition in *cpoA* mutants, their transcription pattern was determined in comparison to the R6 parent strain using an *S. pneumoniae* R6 specific oligonucleotide microarray [21]. Cells were grown under non-competent conditions at pH 6.8 in order to avoid the detection of the complex *com* regulon. Only four gene clusters and one single gene were affected in all three mutants. This included the approximately 3-fold downregulation of a PTS system (spr0276 - spr0282) and an ABC transporter (spr1545 - spr1549), and the 5-7-fold upregulation of two ABC transporters (*vex*, spr0524 - spr0526; spr1558 - spr1560) and spr0307 *clpL* (approximately 4-fold; Additional file 2: Table S3). No effect on PBP genes or genes involved in lipid biosynthesis was apparent.

## Discussion

### Glycolipids in *cpoA* mutants

The two piperacillin-resistant *S. pneumoniae* laboratory mutants P104 and P106, both containing point mutations affecting CpoA production, do not produce detectable amounts of GalGlcDAG, the main glycolipid of this organism. This clearly shows that the glycosysltransferase CpoA of *S. pneumoniae* is essential for the synthesis of the major glycolipid GalGlcDAG *in vivo*, and this could be confirmed by *cpoA* deletion mutants. The data are in agreement with previous *in vitro* studies using extracts of *E. coli* overproducing CpoA [9].

Apparently, the amino acid change in CpoA_P104_ Gly21Val also results in a non-functional protein. Since the mutated protein is still associated with the membrane when cell fractions were probed with anti-CpoA antiserum (Additional file 1: Figure S2), it is possible that the Gly21Val mutation affects protein folding, or its enzymatic function directly or indirectly. In this context it is interesting to note that a missense mutation in *cpoA* has been identified recently in laboratory mutants selected with cefotaxime [22]. The mutation D186Y [listed in the paper as D213Y due to wrong annoation of *cpoA* in the R6 genome [20]] is located within the conserved region of this type of glycosyltransferases, and it would be interesting to study the glycolipid content and phenotype in this mutant. So far, mutations in *cpoA* have not been detected in clinical isolates of *S. pneumoniae*. This might not be surprising since glycolipids are involved in critical cellular functions. On the other hand, the study of laboratory mutants resistant to beta-lactam antibiotics provides a valuable tool to unravel physiological processes related to cell envelope biosynthetic processes.

### Glycolipids and membrane properties

Glycolipids are common in Gram-positive bacteria, and their distribution across the cytoplasmic membrane represents a critical parameter affecting bilayer curvature of the membrane and lipid surface charge densities, thus also membrane-associated functions [23]. Glycolipids in the cell wall-less mycoplasma *Acholeplasma laidlawii* are asymmetrically distributed and mainly external [24]. Clear asymmetry of lipids has also been documented for special membrane systems, such as the purple membrane of the archaebacterium *Halobacterium halobium* where glycolipids were found exclusively in the outer leaflet [25,26], and for the outer membrane of Gram-negative bacteria [27]. It is likely that also in *S. pneumoniae* the two glycolipids are arranged asymmetrically in the membrane and probably predominantly located in the outer leaflet.

Besides glycolipids, membrane proteins can also contribute substantially to the morphology and curvature of membranes [28]. The two GTs of *A. laidlawii*, homologues of Spr0982 and CpoA, have recently been shown to induce membrane vesiculation upon overproduction in *E. coli*[29]. These enzymes are monotopic, i.e. anchored in the membrane cytoplasmic interface by hydrophobic and charge interactions in a SecYEG-independent manner [8,9]. The data of Wikström *et al.*[29] strongly suggest that the GTs themselves are capable of inducing vesiculation, i.e. convex bending of the membrane. This implies some possible consequences when CpoA is absent, i.e. in P106 and in R6ΔcpoA, in that elimination of CpoA itself could affect the curvature of the membrane.

### Phenotypes of *cpoA* mutants

Failure to synthesize GalGlcDAG, the bilayerforming di-glycosyl-glycolipid, must affect the physical properties of the cytoplasmic membrane considerably, consistent with the pleiotropic phenotype associated with *cpoA* mutants. Introduction of the *cpoA* point mutations present in P104 and P106 into the parental R6 strain conferred the same phenotypes, strongly suggesting that no other mutations besides *cpoA* are present in P104 and P106 (not shown). This included higher susceptibility to acidic stress and increased requirement for Mg^2+^ at low pH, as well as reduced lysis rate under lysis inducing conditions. Moreover, an altered proportion of the two pneumococcal phospholipids was observed in the *cpoA* mutants. Whereas cardiolipin is the major phospholipid in the parental R6 strain, all *cpoA* mutants contained a considerable higher amount of phosphatidylglycerol relative to cardiolipin as shown in Figure 3. Interestingly, mutations in the gene encoding the cardiolipin synthase have been identified in cefotaxime resistant laboratory mutants but have not been investigated further [22]. Since GlcDAG, the only glycolipid in *cpoA* mutants, is non-bilayer prone and cardiolipin as well, apparently the cells are capable to regulate the amounts of lipids to ensure sufficient bilayer structure of the cytoplasmic membrane. Cross-regulation in membrane lipid pathways has already been suggested in *B. subtilis* mutants defective in the cardiolipin synthase gene [30]. MIC values of vancomycin or cycloserine inhibiting late and early stages of peptidogylcan synthesis were not affected in *cpoA* mutants, an indication that the cell wall biochemistry is not affected.

Interestingly, *cpoA* mutants were ten-fold more susceptible to bacitracin, which targets the lipid molecule bactoprenol. The *cpoA* mutants expressed an altered transcription profile compared to that of the R6 strain, mainly by genes encoding membrane proteins such as PTS systems or ABC transporters that represent minor components of the bacterial cell. On the other hand, we could not detect significant changes of the protein profile of cytoplasmic or membrane proteins on SDS-polyacrylamide gels, i.e. no major protein components were affected in terms of quantity (not shown). It is conceivable that the transcriptional changes might be an indirect effect of the altered membrane composition. We recently reported that a higher susceptibility to bacitracin was also noted in *S. pneumoniae* containing a mutated ABC transporter [31]. It is possible that the altered lipid composition of the *cpoA* mutants indirectly affects the ABC transporter function and thus bacitracin MIC.

### Glycolipids as anchor molecules in Gram-positive bacteria

Glycolipids represent the membrane anchor of important membrane-bound cell wall polymers in Gram-positive bacteria. They function as the lipid anchor for LTA and also for another class of membrane-associated cell wall glycopolymers, lipoglycans, which seem to replace LTA in the high GC division of Gram-positive bacteria [32,33]. *Listeria* contain the same glycolipids as *S. pneumoniae*, whereas GlcDAG and GlcGlcDAG represent the major glycolipids in *Bacillus*, *Staphylococcus* and *Enterococcus*. However, these species differ in their biosynthetic enzymes. In *Bacillus* and *Staphylococcus*, both glycolipids are synthesized by one single GT YpfP [34-36], whereas two putative GTs are involved in glycolipid biosynthesis in *Listeria*, *Streptococcus* and *Enterococcus*[9,10,37,38]. In this context it is remarkable that the structure of the *cpoA* operon which includes *obg* and several putative small peptide encoding genes is only maintained within *Streptococcus* spp., and that other Gram-positive bacteria contain *cpoA* (plus *spr0982* in case of *Listeria* and *Enterococcus*) and *obg* homologues at distinct positions in the genome. The reason for this is not clear. Several studies revealed that Obg proteins play a role in many important processes, including DNA replication, chromosome segregation, and regulation of stress responses, but their actual function remains unknown [for review, see [19]].

Most of the species mentioned above contain a polyglycerophosphate LTA backbone which is anchored to the di-glycosyl-DAG lipid. Thus, interference of the biosynthesis of this glycolipid severely affects LTA and accordingly cell wall integrity as was shown for mutants in the *S. aureus* GT YpfP [34,35], the (1 → 2)GTs LafA in Listeria, IagA in group B streptococci, and *E. faecalis* BgsA [37-39]. Deletion mutants of *S. aureus ypfP* produced LTA which was probably attached directly to DAG [34,35]. In the GC-rich organism *M. luteus*, dimannosyl-DAG is the lipid anchor of the essential lipomannan cell wall polymer [40]. Therefore, temperature sensitive mutants defective in lipomannan assembly were isolated of *M. luteus*, and one of them (mms1) contained a reduced amount of dimannosyl-DAG whereas the amount of monomannosyl-DAG was increased [41]. The corresponding *M. luteus* gene encoding a putative GT is unknown; according to BLAST analysis, the GT encoded by *mlut_06690* is a likely CpoA homologue.

In contrast to these organisms, the LTA of *S. pneumoniae* is unique in that it includes choline and unusual sugar moieties in its repeating unit which is identical to that of the wall teichoic acid (WTA) [42]. Genetic evidence suggests strongly that the closely related species *S. oralis* and *S. mitis* contain similar TA molecules [43]. Moreover, special choline-binding proteins are associated with the TA molecules, some of which are involved in crucial functions including cell separation [for review, see [44]], probably one of the reasons why LTA and its biosynthetic enzymes are essential in *S. pneumoniae*.

Early studies predicted the LTA lipid anchor to be Glc(β1 → 3)AATGal(β1 → 3)Glc(α1 → 3)DAG where AATGal is 2-acetamino-4-amino-2,4,6-trideoxy-D-galactose [42], but recent data provide evidence that GlcDAG is the more likely anchor molecule [14], i.e. the product of the reaction catalyzed by the GT Spr0982 [10]. Failure to isolate deletions mutants in *spr0982* are in agreement with the essential nature of the *S. pneumoniae* LTA. No effect on choline incorporation into the cell wall was noted in the piperacillin resistant mutants [1], suggesting that teichoic acids seem to be present in similar amounts in mutant cells compared to R6 and that its biosynthesis is not affected by *cpoA* mutations. The estimated number of molecules for LTA and GlcDAG is in the same range of magnitude. LTA constitutes up to 20% of the lipid molecules in the outer leaflet of the cytoplasmic membrane in *S. pneumoniae*[32], and glycolipids represent 34% of the lipids in *S. pneumoniae*[12] with almost one third being GlcDAG [11].

## Conclusions

Here we have shown that CpoA acts as the glycosyltransferase *in vivo* responsible for the biosynthesis of the major glycolipid GalGlcDAG in *S. pneumoniae*. The altered lipid composition of *cpoA* mutants - GlcDAG as the only glycolipid, and a higher proportion of phosphatidylglycerol relative to cardiolipin - affects many membrane related functions and thus results in a pleiotropic phenotype. The question remains why the selection of piperacillin-resistant laboratory mutants P104 and P106 resulted in the isolation of *cpoA* mutations. Since *cpoA* was not affected in another six mutant families selected with cefotaxime, a beta-lactam that induces a tolerant response [2], the *cpoA* mutations are probably related to the highly lytic action of piperacillin. Changes of the physical properties of the membrane by alteration of the lipid composition might be an effective measure to counteract the lytic response induced by beta-lactams and other agents as well.

## Methods

### Bacterial strains, plasmids, oligonucleotides, growth conditions, and transformation

*Streptococcus* strains and plasmids used in this work are listed in Table 1. PCR primers were synthesized at Operon Biotechnologies and are listed in Additional file 2: Table S1. Primers used for sequencing and confirming the correct integration of DNA sections delivered to the *S. pneumoniae* genome and nested primers are not listed. *S. pneumoniae* was grown in C-medium [45] supplemented with 0.2% yeast extract or in Todd Hewitt Broth [THB] (Becton and Dickinson) at 37°C without aeration. For growth on solid surface, D-agar [46] supplemented with 3% defibrinated sheep blood (Oxoid) was used. Growth of *S. pneumoniae* in liquid cultures was monitored by nephelometry (nephelo units [NU]), and doubling time (generation time) estimated from at least three independent experiments. To determine minimal inhibitory concentractions (MICs) of piperacillin, cultures of *S. pneumoniae*, grown in C-medium to a density of 30 NU, were diluted 1000-fold in 0.9% NaCl, and aliquots (30 μl) of the dilutions were spotted on D-agar plates containing piperacillin at concentrations of 0.01 to 0.3 μg/ml using 0.005 μg/ml intervals. MIC values for bacitracin, vancomycin and cycloserine were also determined on D-agar plates using appropriate dilutions of the antibiotic. Antibiotic resistance genes used for chromosomal integrations in *S. pneumoniae* were selected with 2 μg/ml erythromycin (Erm, *ermAB*), 200 μg/ml kanamycin (Kan, *aphIII*), 200 μg/ml streptomycin (Str, *rpsL*), and 3 μg/ml tetracyclin (Tet, *tetM*), respectively. Transformation of *S. pneumoniae* was performed using naturally competent cells as described previously [47]. Transformation efficiency was calculated as the percentage of colonies obtained on the selective medium compared to the colony number on control plates without antibiotic.

**Table 1:** ***S. pneumoniae *** **strains and plasmids**

**Strains**	**Relevant properties**	**Source or reference**
R6	Unencapsulated laboratory strain	[57]
P106	R6 derivative; piperacillin resisant; *cpoA*	[1,7]
P104	R6 derivative; piperacillin resisant; *cpoA*	[1,7]
AmiA9	*rpsL*_A167C_, Str^R^	[51]
R6s	R6 Str^R^, (AmiA9)	This work
R6ΔcpoA	R6s, *rpsL*, Δ*cpoA*, Str^R^	This work
Plasmids		
pTP2	Selection in *S. pneumoniae*: tetracycline 3 μg/ml	
	Selection in *E.coli*: ampicillin 100 μg/ml	GeneBank Nr. EF061140
pTP2PcpoA-ATG21		This work
pTP2PcpoA-ATG1a		This work
pTP2PcpoA-ATG1a		This work

### DNA manipulations

Isolation of plasmid DNA and routine DNA manipulations were carried out by standard methods [48]. PCR products and DNA recovered after restriction endonuclease digestions were purified using the JETquick spin column technique kit. Restriction enzymes and T4 DNA ligase were purchased from Roche Applied Science or New England Biolabs and used according to the manufacturer’s instructions. PCRs were performed using either Goldstar Red *Taq* polymerase (Eurogentec) or iProof High-Fidelity DNA polymerase (Bio-Rad) according to the manufacturer’s instructions. Nucleotide sequencing was performed using the ABI Prism BigDye Terminator Ready Reaction cycle sequencing kit, version 3.1 (Perkin Elmer-ABI). Nucleotide sequences were analyzed by using the CloneManager and Phred/Phrap/Consed software.

### Identification of transcription start site

The start point of *cpoA* transcription was determined by rapid amplification of cDNA ends (5' RACE) as described previously [49] using RNA of *S. pneumoniae* R6 isolated at a culture density of 40 NU. The primer cpoARACE2 was used for reverse transcription of RNA ligated to the RNA adapter, and the nested primer and cpoARACE1 was used for amplification of cDNA (for primers, see Additional file 2: Table S1 and S2).

### Construction of delivery cassettes, plasmids and mutants

To identify the initiation site of *cpoA* translation, fusions of two DNA fragments with the *lacZ* reporter gene were constructed. They contained P_*cpoA*_ (i) together either with two potential start codons (ATG_1_ and ATG_2_ in Figure 1B), (ii) with a mutation in ATG_2_ (ATA), or (iii) with ATG_1_ only. The three fragments were amplified from chromosomal DNA of *S. pneumoniae* R6 by using the primer pairs PcpoA_Eco_f/PcpoA_r2, PcpoA_Eco_f/PcpoABam_r1a and PcpoA_Eco_f/PcpoABam_r1b, cleaved with *Eco*RI and *Bam*HI, and ligated with the *Eco*RI/*Bam*HI-digested translation probe vector pTP2. The desired plasmids, pTP2PcpoA-ATG21, pTP2PcpoA-ATG1a and pTP2PcpoA-ATG1b were isolated after transformation of *E. coli* DH5α and subsequently used to transform *S. pneumoniae* R6; alternatively plasmids were directly transformed into *S. pneumoniae* R6. DNA from Tet^R^ transformants was PCR-amplified and sequenced to confirm the presence of the *lacZ* fusions in the resulting strains R6-PcpoA-ATG21, R6-PcpoA-ATG1a and R6-PcpoA-ATG1b.

In-frame deletions in *cpoA*, *spr0982*, *spr0983*, *obg*, or *spr0985* were constructed via a two-step process in which the central part of the respective gene(s) was first replaced with the Janus cassette [50] that confers a Kan^R^ Str^S^ phenotype in a Str^R^ background. In the second step, the Janus cassette was deleted, thus restoring the original Str^R^ phenotype. The constituents of ‘replacement fragments’ and ‘deletion fragments’ used in the first and second steps of each deletion were amplified from chromosomal DNA of *S. pneumoniae* R6 by using the primer pairs listed in Additional file 2: Table S2. To generate a ‘replacement fragment’, two PCR products of 0.7 to 1 kb (‘upstream’ and ‘downstream fragment’) flanking the desired deletion were joined with the two ends of the Janus cassette either by the use of appropriate restriction sites added to the ends of the respective primers or by overlap extension PCR with nested primers. The ‘replacement fragment’ was used to transform a Str^R^ derivative of *S. pneumoniae* R6 (R6s) obtained by transformation of R6 with chromosomal DNA carrying the AmiA9 resistance marker [51]. In the resulting Kan^R^ Str^S^ transformants, the correct position of the Janus cassette was confirmed by DNA extraction and PCR with appropriate primers. To generate a ‘deletion fragment’ (containing the desired deletion), the respective ‘upstream’ and ‘downstream fragments’ were directly joined with each other either by the use of appropriate restriction sites added to the primers or by overlap extension PCR with nested primers. The ‘deletion fragment’ was used to transform a derivative of R6s carrying the Janus cassette at the site of the desired deletion. DNA from transformants displaying a Kan^S^ Str^R^ phenotype was PCR-amplified and sequenced to confirm the presence of the deletion in the resulting mutant.

### Determination of β-galactosidase activity

Preparation of cell extracts from cultures of *S. pneumoniae*, grown to a density of OD_600_ = 0.8 in C-medium, and determination of specific β-galactosidase activities were performed as described [52].

### Lipid extraction and analysis

Lipids were extracted from *S. pneumoniae* essentially as described [53]. Briefly, cells harvested by centrifugation of liquid cultures grown to a density of about 70 NU were resuspended in 0.8 ml H_2_O per gram wet weight and subsequently mixed with 3 ml of chloroform/methanol (1:2) per gram wet weight. After gentle agitation for 2 h at 4°C, chloroform (1 volume) and H_2_O (1 volume) were added and mixed. The samples were centrifuged at 4,000 × g and 4°C for 5 min, the organic phases were recovered, mixed with 1 volume of H_2_O equilibrated with chloroform/methanol (1:2), and centrifuged as before. Recovered organic phases were completely evaporated, and the remainders were dissolved in 50 to 100 μl of chloroform/methanol (80:15). Glycolipids were separated by one-dimensional thin layer chromatography in chloroform/methanol/acetic acid (80:15:8) on silica gel G plates (0.025 mm; Merck). For visualization the plates were sprayed with 1-naphthol (3.2% w/v in methanol/H_2_SO_4_/H_2_O = 25:3:2) and heated at 110°C for 10 min. GalGalDAG (Sigma) and GlcDAG were used as standards. Phospholipids were separated on two-dimensional thin layer chromatography (first dimension: CHCl_3/_MeOH/H_2_O = 65:25:4; second dimension: CHCl_3_/AcOH/MeOH/H_2_O = 80:14:10:3) and stained with 1.3% molydbenum oxide in 4.2 M sulfuric acid (Molybdenum Blue spray reagent, Sigma-Aldrich). Spots were assigned according to the reference lipid phosphatidylglycerol (Sigma) and the pattern described elsewhere for phospholipids [42].

### Immunological detection of CpoA

*S. pneumoniae* cells were grown to mid-exponential growth phase (80 NU), harvested by centrifugation (9,000 rpm, 15 min, 4°C, Beckman centrifuge J2-21), and washed once with 20 mM sodium phosphate buffer pH 7.2. Pellets were resuspended in 180 μl sodium phosphate buffer and mixed with 500 mg glass beads per 100 mg wet weight, followed by disruption in a cell mill (Vitrogen-Zellmühle Typ VI-4, Edmund Bühler GmbH) for 20 min. All further steps were carried out on ice. Glass beads were removed by centrifugation for 6 min (14,000 rpm, 4°C, Hermle Z513K centrifuge). Membranes were separated from cytoplasmic proteins by ultracentrifugation (Beckman centrifuge, TLA 100.4 rotor) for 2 h at 60,000 rpm and 4°C. Pellets were resuspended in half of the volume of the supernatant, and fractions stored at −80°C. For SDS polyacrylamide gel electrophoresis, 3 μl per fraction were used. Western blotting was performed as described previously [54] and CpoA was visualized using a 1:10,000 dilution of rabbit antiserum raised against a purified CpoA-derivative as described [7].

### Microarray-based transcriptome analysis

Extraction of total RNA from exponentially growing *S. pneumoniae* cultures (40 NU), reverse transcription of RNA into labeled cDNA, prehybridization, hybridization, slide washing, scanning, and analysis of the data were performed as described previously [55]. For each strain, data sets from at least four hybridizations were used for normalization and statistical analysis. Only data which showed *P* values below 10^-4^ in a paired *t* test, and relative changes in the transcript amount of greater than threefold were considered further. The oligonucleotide microarray covering genes and intergenic regions of *S. pneumoniae* R6/TIGR4 has been described [21].

### Accession number

*S. pneumoniae* R6/TIGR4 oligonucleotide microarray: ArrayDesign R6/TIGR4 ArrayExpres accession number A-MEXP-1846.

## Availability of supporting data

The data sets supporting the results of this article are included within the article and its additional files.

## Abbreviations

aa: Amino acids; bp: Base pairs; GalGlcDAG: 1,2-diacyl-3–*O*–[α-D-glucopyranosyl-(1 → 2)-*O*–α-D-galactopyranosyl]-*sn*-glycerol; GlcDAG: 1,2-diacyl-3-*O*–(α-D-glucopyranosyl)-*sn*-glycerol; GT: Glycosyltransferase; LTA: Lipoteichoic acid; NU: Nephelo units.

## Competing interests

- In the past five years have you received reimbursements, fees, funding, or salary from an organization that may in any way gain or lose financially from the publication of this manuscript, either now or in the future? Is such an organization financing this manuscript (including the article-processing charge)? no- Do you hold any stocks or shares in an organization that may in any way gain or lose financially from the publication of this manuscript, either now or in the future? No

- Do you hold or are you currently applying for any patents relating to the content of the manuscript? Have you received reimbursements, fees, funding, or salary from an organization that holds or has applied for patents relating to the content of the manuscript? No

- Do you have any other financial competing interests? No

Non-financial competing interests

- Are there any non-financial competing interests (political, personal, religious, ideological, academic, intellectual, commercial or any other) to declare in relation to this manuscript? No

## Authors’ contributions

MM, CV and JE carried out the molecular genetic studies and phenotypic analyses; MM carried out immunoassays and lipid chromatography. RH, BH and PM conceived of the study; RH and BH participated in its design and coordination and helped to draft the manuscript. All authors read and approved the final manuscript.

## Supplementary Material

Additional file 1: Figure S1 Phospholipids in *S. pneumoniae* R6. Lipids were extracted and separated by two dimensional TLC. 1.D and 2.D: first and second dimension (first dimension: CHCl_3_/MeOH/H_2_0 = 65:25:4; second dimension: CHCl_3_/AcOH/MeOH/H_2_0 = 80:14:10:3). Phospholipids were visualized by spraying with Molybdenum Blue spray reagent. PG: phosphatidylgylcerol; CL: cardiolipin. Standards: PG, 0.3 μMol; CL, 0.17 μmol. **Figure S2.** Membrane association of CpoA. Membrane (m) and cytoplasmic proteins (s) were separated by SDS-PAGE followed by immunostaining with anti-CpoA antiserum (see Methods for detail). Closed arrows indicate the position of CpoA in the membrane fractions of *S. pneumoniae* R6 and P104, the open arrow shows the absence of CpoA in R6ΔcpoA. M: marker proteins. Click here for file

Additional file 2: Table S1 Primers. **Table S2.** PCR primer pairs used for the construction of in-frame deletions ^1^. **Table S3.** Altered transcription profiles in *cpoA* mutants. Click here for file
