# Supplementary figures and images for: Altered lipid composition in Streptococcus pneumoniae cpoA mutants
Source: BMC Microbiol. 2014 Jan 20;14:12. doi: 10.1186/1471-2180-14-12 (PMC3901891; doi:10.1186/1471-2180-14-12)

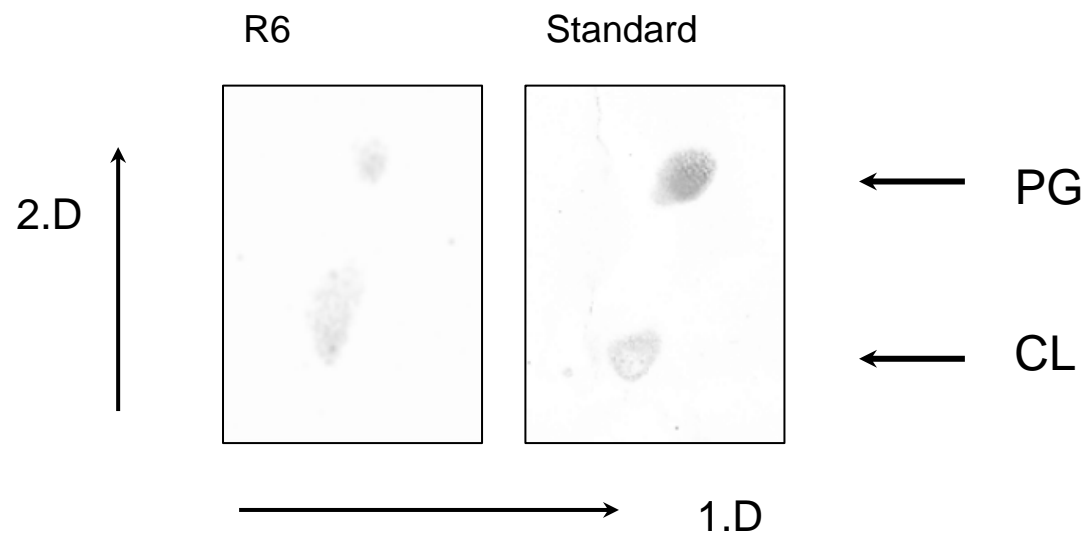

Supplement Fig. 1

Meiers et al.

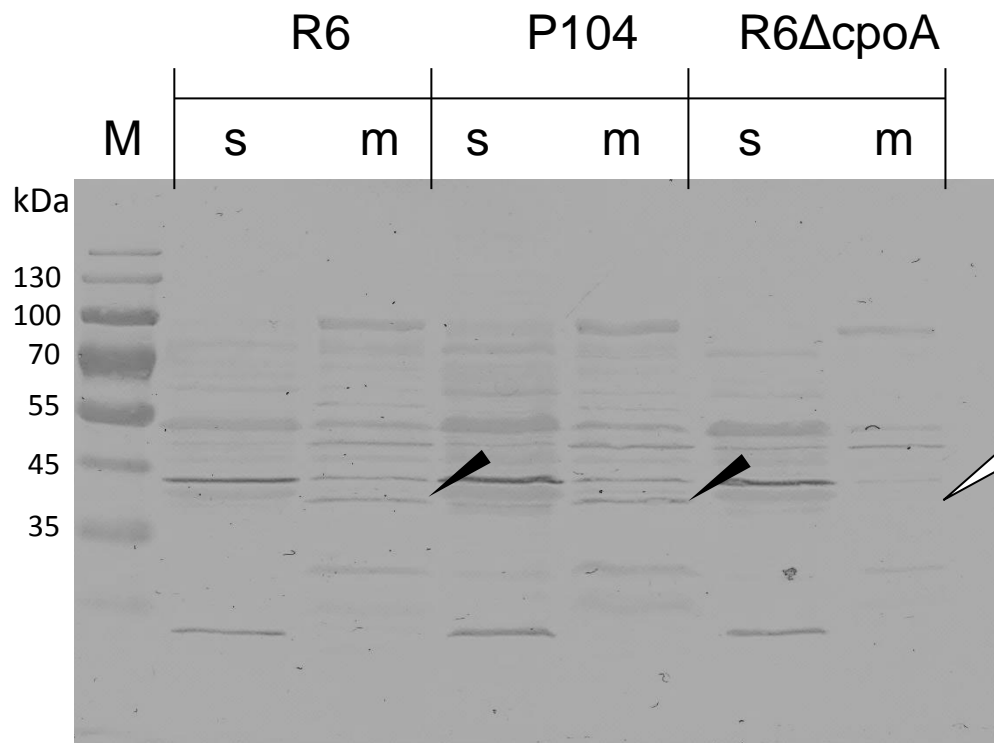

Supplement Fig. 2

Meiers et al.

Supplement: Additional file 1: Figure S1 — Phospholipids in S. pneumoniae R6. Lipids were extracted and separated by two dimensional TLC. 1.D and 2.D: first and second dimension (first dimension: CHCl3/MeOH/H20 = 65:25:4; second dimension: CHCl3/AcOH/MeOH/H20 = 80:14:10:3). Phospholipids were visualized by spraying with Molybdenum Blue spray reagent. PG: phosphatidylgylcerol; CL: cardiolipin. Standards: PG, 0.3 μMol; CL, 0.17 μmol. Figure S2. Membrane association of CpoA. Membrane (m) and cytoplasmic proteins (s) were separated by SDS-PAGE followed by immunostaining with anti-CpoA antiserum (see Methods for detail). Closed arrows indicate the position of CpoA in the membrane fractions of S. pneumoniae R6 and P104, the open arrow shows the absence of CpoA in R6ΔcpoA. M: marker proteins. [file 1471-2180-14-12-S1.pdf]
