# Supplementary material for: Altered lipid composition in Streptococcus pneumoniae cpoA mutants
Source: BMC Microbiol. 2014 Jan 20;14:12. doi: 10.1186/1471-2180-14-12 (PMC3901891; doi:10.1186/1471-2180-14-12)
Supplement: Additional file 2: Table S1 — Primers. Table S2. PCR primer pairs used for the construction of in-frame deletions 1. Table S3. Altered transcription profiles in cpoA mutants. [file 1471-2180-14-12-S2.doc]

Table S1. Primers

Primer Sequence

0982down_f-del1 *GCGACCAGTATTCGAACC*TATCTTCCGCAACAGGTGGTTGCTGTACC

0982down_f-rps *CGTCCAAAAGCATAAGGAAAGGGGCCC*TATCTTCCGCAACAGGTGGTTGCTGTACC

0982down_r TGTTAAAACCTTGCCAGTCTCCGCATCACG

0982up_f TGTGCATTTCTTGCCAGCTACACTTGAGGG

0982up_fff GAGGATTTGGTAGCAGCTGGTATTCCACG

0982up_r-kan *CGGATCCGATCCATTTCCTCTGGAATAGG*GGTTCGAATACTGGTCGCAACACCAGAAAC

0983down_f-del3 *GTGATAAAAAGATTGCGGGT*GCACCAGTAGCAACTGACTATTGAAAACTAGC

0983down_f-rps *CGTCCAAAAGCATAAGGAAAGGGGCCC*GCACCAGTAGCAACTGACTATTGAAAACTAGC

0983down_r TCCATGAGGCGAAGATTGTAAGACTCCAGC

0983up_f GCCGAGTATGGTCAAGTATCTGGTTAGAGG

0983up_r-del3 *TAGTCAGTTGCTACTGGTGC*ACCCGCAATCTTTTTATCACGACCGCTTCTC

0983up_r-kan *CGGATCCGATCCATTTCCTCTGGAATAGG*ACCCGCAATCTTTTTATCACGACCGCTTCTC

0985down_fApa *agtgggccc*CGTGCCTTGAGATTGGCACGAACTACAAAG

0985down_fBam *cgcggatcc*CGTGCCTTGAGATTGGCACGAACTACAAAG

0985up_f GGTGCTGAGGACCTTAGAGTTCGAGTACC

0985up_rBam *cgcggatcc*GGCGGAAACAAAATTACCATCCGCATCTCG

cpoA_rev *CGCGGATCC*AATTTCTCTGATAAGGTATGC

cpoA5'_f TGAGAAAGTAGCAGGACAGGGAGTTTCAGG

cpoALr *GCGGATCC*CTCTTCTTGTGGTAGAGGATG

cpoARf *TAGGGCCC*GCGTGATTTAGATCTCTATAAGG

cpoARf3 *GCGGATCC*GAAGCTGCGAGTTGTGAGGC

cpoAseq1 GCGCACACTTGAAGATGTGG

cpoAseq5 TCCTGCTTGATTTCCGGACG

erm18_f *CGAATTC*AGCAAAGAATGGCGGAAACG

erm18_r *GCCTCGAG*CTTTAGTAACGTGTAACTTTCC

Janus_f CCTATTCCAGAGGAAATGGATCGGATCCG

Janus_r GGGCCCCTTTCCTTATGCTTTTGGACG

obg3'_r ACTCAAATTTACCAATGCGGACCAAATCCC

obg3up_rBam *cgcggatcc*GCCACCATTACCAGCCTTGACCTTAATCTTAGC

obg5'_f AGCTAAGATTAAGGTCAAGGCTGGTAATGG

obgdown_fApa *agtgggccc*GCTAAAGATGGGGATTTGGTCCGCATTGG

obgdown_fSal *tagtgggtcgac*GCTAAAGATGGGGATTTGGTCCGCATTGG

obgdown_r TCACTTTCTTCGCTACGATAGAGCCGTTCG

obgup_f CCTAGTGAGACGGCTCTTTACTATAAAGCG

obgup_rSal *tagtgggtcgac*GCCACCATTACCAGCCTTGACCTTAATCTTAGC

PcpoABam_r2 *CCGCGGATCC*AACATATTGATGCGTAATTTCTTTTTCTCCATAAC

PcpoABam_r1a *CGCGGATCC*TTGATGCGTAATTTCTTTTTCTCCATAACTACTATTATATC

PcpoABam_r1b *CGCGGATCC*CCCTGTCCTGCTACTTTCTCACTTGAACTCAATATATTG

PcpoAEco_f *CCGGAATT*CTCTAGTCTCTCCTTTCTTTTGCTGATTTTATTC

spr09823'_r TCCAATAGTCTCTCATACTGATCAACTGTG

spr09825'_f TACAGATACCTATTTTCCTCAGGTTTCTGG

spr0982up_r-del1 *ACCTGTTGCGGAAGATAC*GGTTCGAATACTGGTCGCAACACCAGAAAC

spr09833'_r GCGATAATCCATAAAATAATGTAAGCTACAATTCC

spr09835'_f TGATAAAAAGATTGCGGGTGTTTGTGCTGG

spr09853'_r CTTTGTAGTTCGTGCCAATCTCAAGGCACG

^1^ Nucleotides printed in italics were added to introduce restriction enzyme cleavage sites (underlinded) or overlaps with other DNA fragments

Table S2. PCR primer pairs used for the construction of in-frame deletions ^1^

Deletion Generation of 'replacement fragments' ^2^ Generation of 'deletion fragments' ^3^

upstream fragment downstream fragment Janus cassette upstream fragment downstream fragment

Δ*cpoA* cpoAseq1 cpoARf (*Apa*I) Janus_f cpoAseq1 cpoARf3 (*Bam*HI)

cpoALr (*Bam*HI) cpoAseq5 Janus_r cpoALr (*Bam*HI) cpoA_rev

Δ*spr0982* 0982up_f 0982down_f-rps (*rps*) Janus_f 0982up_f 0982down_f-del1 (up)

0982up_r-kan (*aph*) 0982down_r Janus_r spr0982up_r-del1 (down) 0982down_r

Δ*spr0983* 0983up_f 0983down_f-rps (*rps*) Janus_f 0983up_f 0983down_f-del3 (up)

0983up_r-kan (*aph*) 0983down_r Janus_r 0983up_r-del3 (down) 0983down_r

Δ*obg* obgup_f obgdown_fApa (*Apa*I) Janus_fSal(*Sal*I) obgup_f obgdown_fSal (*Sal*I)

obgup_rSal (*Sal*I) obgdown_r Janus_r obgup_rSal (*Sal*I) obgdown_r

Δ*spr0985* spr0985up_f spr0985down_fApa (*Apa*I) Janus_f spr0985up_f spr0985up_rBam (*Bam*HI)

spr0985up_rBam (*Bam*HI) obgdown_r Janus_r spr0985down_fBam (*Bam*HI) spr0985down_r

^1^ Nucleotide sequences of individual primers. Specifications in parentheses refer to restriction sites, overlaps with the *aphIII*-proximal (*aph*) or the *rpsL*-proximal (*rps*) ends of the Janus cassette, or overlaps with the corresponding downstream (down) or upstream (up) fragments, added to the 5' ends of the respective primers

^2^ Primers used to amplify the constituents of 'replacement fragments' consisting of the Janus cassette flanked by DNA fragments ('upstream fragment', 'downstream fragment') corresponding to the upstream and downstream sequences of the desired deletions

^3^ Primers used to amplify the constituents of 'deletion fragments' carrying the desired deletions flanked by the corresponding upstream and downstream sequences ('upstream fragment', 'downstream fragment')

Table S3. Altered transcription profiles in *cpoA* mutants

Locus Gene P104 P106 Δ*cpoA* Product

spr0276 *bglA* 0,16 0,11 0,04 6-phospho-beta-glucosidase

spr0277   0,15 0,15 0,05 Conserved hypothetical protein

spr0278 0,18 0,27 0,21 Phosphotransferase system sugar-specific EII component

spr0279 *bglG* 0,22 0,26 0,2 Transcription antiterminator BglG family

spr0280 0,33 0,2 0,21 Phosphotransferase system sugar-specific EII component

spr0281   ^1^ 0,14 ^1^ Hypothetical protein

spr0282 ^1^ 0,24 0,19 Phosphotransferase system sugar-specific EII component

spr0307 *clpL* 3,97 3,35 6,16

spr0524 *vex1* 8,71 6,04 7,33 ABC transporter membrane-spanning permease - Pep export

spr0525 *vex2* 5,16 3,71 ↑ ABC transporter ATP-binding protein - Pep export

spr0526 *vex3* 7,53 4,36 3 ABC transporter membrane-spanning permease - Pep export

spr1545   0,25 0,32 ^2^ Hypothetical protein

spr1546 0,25 ↓ 0,28 ABC transporter ATP-binding protein - unknown substrate

spr1547 0,21 ↓ 0,19 Hypothetical protein

spr1548   0,32 ↓ 0,15 Hypothetical protein

spr1549   0,27 ↓ 0,19 Hypothetical protein

spr1558   ↑ 3,57 4,16 Conserved hypothetical protein

spr1559 3,37 4,77 5,19 ABC transporter ATP-binding protein - unknown substrate

spr1560 ↑ 3,9 4,81 Hypothetical protein

^1^ not detected

^2^ not significant

arrows indicate higher or lower signals compared to R6 but below the three-fold significance threshold

Figure S1. Phospholipids in *S. pneumoniae* R6

Lipids were extracted and separated by two dimensional TLC. 1.D and 2.D: first and second dimension (first dimension: CHCl_3_/MeOH/H_2_0 = 65:25:4; second dimension: CHCl_3_/AcOH/MeOH/H_2_0 = 80:14:10:3). Phospholipids were visualized by spraying with Molybdenum Blue spray reagent. PG: phosphatidylgylcerol; CL: cardiolipin. Standards: PG, 0.3 µMol; CL, 0.17 µmol.

Figure S2. Membrane association of CpoA.

Membrane (m) and cytoplasmic proteins (s) were separated by SDS-PAGE followed by immunostaining with anti-CpoA antiserum (see Methods for detail). Closed arrows indicate the position of CpoA in the membrane fractions of *S. pneumoniae* R6 and P104, the open arrow shows the absence of CpoA in R6ΔcpoA. M: marker proteins.
